# Prevalence of Depression and Related Factors among Patients with Chronic Disease during the COVID-19 Pandemic: A Systematic Review and Meta-Analysis

**DOI:** 10.3390/diagnostics12123094

**Published:** 2022-12-08

**Authors:** Rubén A. García-Lara, Nora Suleiman-Martos, María J. Membrive-Jiménez, Victoria García-Morales, Miguel Quesada-Caballero, Isabel M. Guisado-Requena, José L. Gómez-Urquiza

**Affiliations:** 1 UGC Orgiva, Granada-South Health Management Area, Andalusian Health Service, Calle La Madre s/n, Lanjarón, 18420 Granada, Spain; 2 Nursing Department, Faculty of Health Sciences, University of Granada, Av. de la Ilustración, 60, 18016 Granada, Spain; 3 Red Cross Nursing Center, University of Sevilla, Av. la Cruz Roja, 41009 Sevilla, Spain; 4 Department of Biomedicine, Biotechnology and Public Health, Faculty of Medicine, University of Cadiz, Pl. Falla, 9, 11003 Cadiz, Spain; 5 UGC La Caleta Granada-Metropolitano, Andalusian Health Service, Av. del Sur, 11, 18014 Granada, Spain; 6 Nursing Department, Faculty of Health Sciences, University of Castilla la Mancha, 02006 Albacete, Spain; 7 Nursing Department, Faculty of Health Sciences, University of Granada, Cortadura del Valle s/n, 51001 Ceuta, Spain

**Keywords:** COVID-19, chronic disease, depression, prevalence, related factors

## Abstract

The management of chronic diseases in the midst of the COVID-19 pandemic is especially challenging, and reducing potential psychological harm is essential. This review aims to determine the prevalence of depression during the COVID-19 pandemic in patients with chronic disease, and to characterize the impacts of related factors. A systematic review was conducted in accordance with the Preferred Reporting Items for Systematic Reviews and Meta-Analyses (PRISMA) guidelines. The meta-analysis was performed using StatsDirect software. The review identified 33 articles with a total of 50,905 patients with chronic diseases. Four meta-analyses were performed to estimate the prevalence of depression. In diabetic patients, the prevalence ranged from 17% (95% CI = 7–31) (PHQ-9) to 33% (95% CI = 16–51) (PHQ-8); in obese patients, the prevalence was 48% (95% CI = 26–71); and in hypertensive patients, the prevalence was 18% (95% CI = 13–24). The factors significantly associated with depression were female sex, being single, deterioration in the clinical parameters of diabetes, a decrease in self-care behavior, reduced physical activity and sleep time and fear of contagion. The COVID-19 pandemic has significantly increased levels of depression among persons with chronic disease. Pandemics and other emergency events have a major impact on mental health, so early psychological interventions and health management policies are needed to reinforce chronic patients’ physical and mental health.

## 1. Introduction

Since the emergence of COVID-19 in December 2019, the world’s population and healthcare systems have been exposed to enormous challenges [1]. One aspect of this is that fear of infection and the subsequent need for social distancing and/or isolation have strongly impacted the diagnosis and treatment of diseases. In up to 50% of cases, patients’ health status has worsened and 17% have developed a new disease [2]. Moreover, the temporary suspension of medical services during confinement reduced the number of chronic disease diagnoses by up to 26% [3].

The pandemic restrictions have severely affected the general health of the population, and patients with chronic diseases are at particular risk [4]. Persons in this group suffer high levels of morbidity and mortality, are more likely to present adverse outcomes, and often respond more poorly to COVID-19 infection [5]. In addition to these consequences, the quarantine measures imposed and the saturation of the healthcare system during successive waves of infection have provoked discontinuities in the care provided to patients with pre-existing chronic pathologies. Thus, up to 54% of chronic patients have suffered negative repercussions in the treatment and management of their condition [2].

The negative impact on health is reflected not only in clinical parameters, but also in terms of mental health. Depression, loneliness and anxiety are among the most common causes of care demand in primary care [6] and the pandemic has further increased the demand for these services [7,8]. Visits for anxiety and depression were the most common reasons for a virtual visit (90.6% virtual visits) [7]. Before the pandemic, 7.9% of primary care visits were due to depression, but this figure then rose to 8.5% [7]. This may suggest that rates of depression increased since the onset of the COVID-19 pandemic [9], but this is not the case; as diagnoses of common conditions decreased during the pandemic, a large number of patients’ conditions remained undiagnosed, meaning that there was no increase in depression diagnoses made by primary care physicians [10,11].

Comorbidities such as depression can make proper control of the disease difficult [12]. Specifically, patients may experience greater depression following sustained changes in living conditions due to social distancing, the need for isolation and the fear provoked by successive waves of contagion [13]. In these circumstances, the number of patients with physical, mental or emotional health needs is expected to increase significantly, a situation that may become chronic [7].

Although studies have been carried out to determine the prevalence of depressive symptoms among older adults, the results obtained have been inconsistent, with reported levels ranging from 18.6% [14] to 40% [15]. In addition, there are significant deficiencies in the mental health care provided for persons with chronic disease. In a recent meta-analysis of patients with different health conditions, mainly older adults infected by COVID-19, older adults who were generally healthy and older adults with chronic disease reported a prevalence of depression of 61% [16], but to our knowledge, no comparable study has been undertaken specifically to determine the levels of depression suffered by patients with chronic disease.

In this respect, an analysis of the current situation, including the prevalence of depression in chronic patients, the follow-up attention provided and related factors, would facilitate the development and application of suitable intervention strategies. An important aspect of this during the present COVID-19 pandemic scenario is the need to screen chronic patients for depression, in order to implement health policies aimed at optimizing their physical and mental health [17]. The aim of this systematic review and meta-analysis is to determine the prevalence of depression among patients with chronic disease during the COVID-19 pandemic, as well as related factors.

## 2. Methods

### 2.1. Design

This systematic review and meta-analysis was performed in accordance with the PRISMA guidelines [18] (see PRISMA checklist in Supplementary Material). The protocol was registered in PROSPERO (International Prospective Register of Systematic Reviews) with the registration number CRD42021298329.

### 2.2. Search Methods

A comprehensive literature search on the databases EMBASE (Ovid), the Cumulative Index to Nursing and Allied Health Literature (CINAHL) (EBSCO), Medline (Ovid), SciELO (BIREME Virtual Health Library) and Scopus (Elsevier) was performed during October 2022 with no restrictions on language or year of publication. The search terms were: “(diabetes OR hypertension OR hyperlipidemia OR dyslipidemia OR obesity OR chronic disease OR chronic illness OR chronically ill OR non-communicable diseases) AND (SARS-CoV-2 OR coronavirus OR COVID-19) AND (depression OR depressive disorder)”. The PICO (Population, Intervention, Comparison, Outcome) strategy was applied to answer the question: What is the prevalence of depression among patients with chronic diseases during the present COVID-19 pandemic, and what other interrelated factors are present?

### 2.3. Search Outcomes

The articles included in the analysis were those that met the following inclusion criteria: (1) original quantitative studies conducted during the COVID-19 outbreak, (2) patients with chronic disease (diabetes type 1 and type 2, hypertension, hyperlipidemia, dyslipidemia, or obesity), (3) analyses of depression levels (percentages, mean or median), (4) the use of a validated measurement tool for depression and (5) analyses of factors related to depression. There was no restriction on language or date of publication.

Studies presenting the following characteristics were excluded from the analysis: (1) review articles, letters to editors, conference abstracts and case reports; (2) those based on participants with serious cognitive/neurological impairment, severe mental illness or mental/physical disability; (3) studies that did not investigate the diagnosis of depressive disorders during the COVID-19 pandemic; (4) studies considering chronic pathologies but lacking specific depression data for each of them; (5) those based on data evaluated using an unvalidated scale.

### 2.4. Quality Appraisal

The degree of bias present in the observational studies considered was determined using the STROBE (Strengthening the Reporting of Observational Studies in Epidemiology) guidelines [19].

Quality assessment was performed to decide whether or not each study would included in the review. The quality of the studies was assessed according to the recommendations of the OCEBM (Oxford Center for Evidence-Based Medicine), with respect to the levels of evidence and grades of recommendation [20] (Table 1). Both the risk of bias and the quality of evidence were assessed by two reviewers working independently.

### 2.5. Data Abstraction

**Figure 1:**
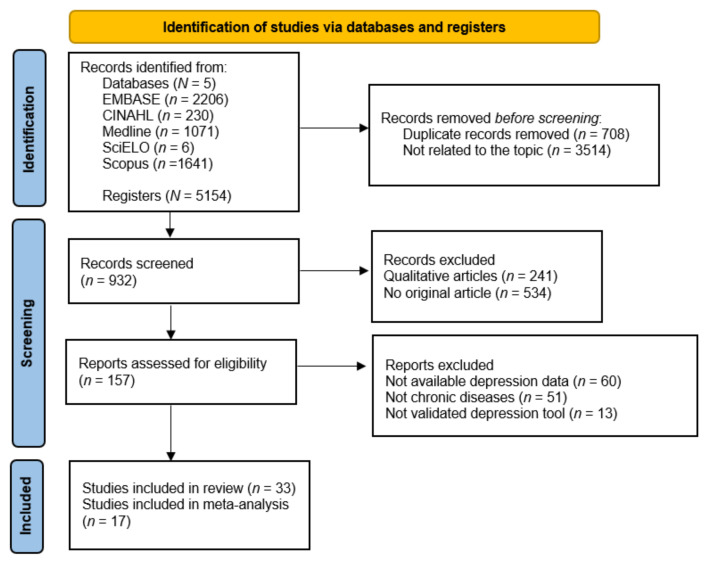
Flow diagram of the publication search process.

The initially identified studies were then assessed for inclusion by two independent reviewers (R.G.-L., N.S.-M.), who first analyzed the titles and abstracts, and then, the full texts, according to their accordance or otherwise with the inclusion criteria (see Figure 1). Any disagreements in this respect were resolved by consulting a third reviewer (J.L.G.-U.).

In this selection task, the necessary data were extracted and recorded in a spreadsheet by two of the authors (M.J.M.-J., V.G.-M.). Any disagreement was resolved via consultation with a third author (J.L.G.-U). Among other data, the following variables were extracted from the articles included: (1) author, year of publication, country, (2) study design and period, (3) size and characteristics of the sample, (4) setting of the study, (5) measuring instruments or depression scale used, (6) type of chronic disease, (7) levels of depression (percentage, mean or median) and (8) related factors (Table 1 and Table 2).

### 2.6. Synthesis/Data Analysis

For the systematic review, the data were extracted via a descriptive analysis, and then, classified in a data table. Levels of depression were defined according to different scales for depression and cut-offs. Regarding severity, the nine-item version of the Patient Health Questionnaire (PHQ-9) and eight-item version (PHQ-8) comprise five categories, where a cut-off point of 0–4 indicates no depression, 5–9 mild depressive symptoms, 10–14 moderate depressive symptoms, 15–19 moderately severe depressive symptoms, and 20–27 severe depressive symptoms [21,22]. For the two-item Patient Health Questionnaire—2 (PHQ-2), the severity score ranges from 0–6, where a cut-off score of 3 or more indicates major depression [23]. For the Hospital Anxiety and Depression Scale (HADS) depression severity is defined as: 0 to 7, no depression; 8 to 10, mild depression; 11 to 14, moderate depression; 15 to 21, severe depression. A score of ≥11 was considered a clinically significant disorder [24]. For the Stress, Anxiety and Depression Scale (DASS-21) the following severity scale is used: normal, 0–9; mild, 10–13; moderate, 14–20; severe, 21–27; and extremely severe, more than 27 [25]. The score values of the rest of the depression scales are shown in Table 1.

The meta-analysis was applied to all the studies that presented sufficient statistical data, using the same scale or instrument in each case for measuring depression, since the inclusion of different ones, producing different scores, would not allow the results to be integrated.

Heterogeneity was analyzed using the I^2^ index, which represents the percentage of variation attributable to statistical heterogeneity. Fixed or random-effects analysis was used depending on the heterogeneity of the sample. If I^2^ was >50%, random-effects analysis was used [26]. Publication bias was assessed using Egger’s linear regression, and sensitivity analysis was also performed.

Four meta-analyses were performed to estimate the prevalence of depression and the corresponding confidence interval. Two random-effects meta-analyses were focused on depression in diabetic patients, using PHQ-9 and PHQ-8. One random-effects meta-analysis considered the presence of depression in obese patients, measured using the PHQ-9 questionnaire, and one fixed-effects meta-analysis measured depression in hypertensive patients using the HADS. All statistical calculations were performed using StatsDirect software (3.0). The statistical program evaluated the weighting of the influence of each study on the result of the meta-analysis.

## 3. Results

### 3.1. Characteristics of the Studies Included

The initial literature search obtained 5154 results. After reading the full text of each one and determining its accordance with the inclusion criteria, 33 articles were included in the final analysis. The complete study search and selection process is shown in Figure 1.

Among the studies extracted by the literature search, 72.7% were cross-sectional and the rest were longitudinal. These studies represented a total sample size of 50905 patients with chronic disease. Eight studies had been conducted in WHO’s Eastern Mediterranean region, five in Turkey, five in the USA, three in Brazil, two each in Italy, Germany, Korea and the UK, and the rest in other countries (see Table 1).

To measure depression, 13 of the studies used the PHQ-9 questionnaire, while the others used the two- or eight-item versions (PHQ-2 and PHQ-8, respectively). The other most used depression measurement tools were the Hospital Anxiety and Depression Scale (HADS) and the Stress, Anxiety and Depression Scale (DASS-21) (Table 1).

The study data were collected in various settings, including hospital units, outpatient clinics and online surveys. All studies presented an adequate level of quality, and none were excluded for deficiencies in this respect. The characteristics and findings of the studies are shown in Table 1.

### 3.2. Levels of Depression and Related Factors in Patients with Diabetes

The mean levels of depression found in diabetic patients ranged from minimal [27,28,29,30], to mild [31,32,33,34,35] to moderate [36,37] (Table 1).

Factors reported to be associated with the presence of depressive symptoms in diabetic patients during the COVID-19 pandemic included female gender [30,32,36,38,39,40,41,42], being single [38,41], the absence of religious faith [41], lower education level [38], smoking [40], a history of anxiety and/or depression [41], lower income [41] and part-time work or remote study [41]. In relation to age, some authors indicate that younger age is a predisposing factor [28,32,38] while others found no significant relationship [39] (Table 2).

The clinical predictors of diabetes found to be related to higher levels of depression included high HbA1c [38,39,43,44], low daily time-in-range blood glucose [38,45], type 2 diabetes [27,32], and diabetes duration of >5 years [38]. According to Chao et al. (2021), obesity was a predisposing factor for depression, but Alaqeel et al. (2021) obtained no significant results in this respect (Table 2).

Other factors that have been related with depression include difficulty accessing diabetes supplies [38,43], changes in diabetes self-care behavior and diet [38,46,47], and fear of acquiring the coronavirus infection [28,29,33,38,40,48] (Table 2).

An association between depression and a lower quality of life among diabetic patients was also reported [34,37], as were reduced physical activity and sleep time as relevant factors [46,47] (Table 2).

Among the studies that analyzed depressive symptoms before and after the initial COVID-19 outbreak [28,42,48], two of them [28,42] found relatively stable levels of depression. However, data were collected during the first wave during an early stage of the pandemic, and the time of study participation might impact self-reported data. Additionally, the data of the questionnaires and telephone interviews may had led to possible selection and self-report biases.

**Table 1:** Characteristics of the included studies (*n* = 33).

Author, Year, Country	Study/Period	Sample	Setting	Depression Screening Tool	Type of Chronic Disease	Depression Mean (SD)/Median (IQR)/Prevalence	EL/RG
Abdelghani et al., [34] 2021, Egypt	Cross-sectional June–September 2020	N = 200 Mean age: 48.4 (13.7) Female: 63% Mean duration of DM: 6.2 (5.3) years	Endocrinology outpatient clinic	HADS (range: 0–21, score of ≤ 7: normal, 8–10: mild, 11–14: moderate, ≥15: severe depression, cut-off score for depression ≥11)	T1D T2D	8.9 (4.5)	2b/B
Abdoli et al., [38], 2021, US, Brazil, and Iran	Cross-sectional April–June 2020	N = 1788 US (*n* = 1099) ^a^ Brazil (*n* = 477) ^b^ Iran (*n* = 212) ^c^ Age > 18 years Female: 78.28%	Web-based survey	PHQ-8 (0–4: minimal, 5–9: mild, 10–14: moderate, 15–19: moderately severe, 20–27: severe depressive symptoms, cut-off score for depression >10)	T1D	Moderate–severe US: 26.4% ^a^ Brazil: 52.8% ^b^ Iran: 60.9% ^c^	2b/B
Ahmed et al., [49], 2022, Sudan	Cross-sectional	DM *n* = 89 Hypertension *n* = 86 Age > 24 years	Primary healthcare centers	PHQ-9 (0–4: minimal, 5–9: mild, 10–14: moderate, 15–19: moderately severe, 20–27: severe depressive symptoms, cut-off score for depression >10)	DM Hypertension	*DM*Minimal: 33.33% Mild: 32.22% Moderate: 14.44% Moderate–severe: 8.89% Severe: 10% *Hypertension*Minimal: 32.56% Mild: 26.74% Moderate: 4.42% Moderate–severe: 11.63% Severe: 4.65%	2b/B
Ajele et al., [35], 2022, Nigeria	Cross-sectional April–July 2021	N = 223 Mean age: 53.26 (11.05) Female: 26%	Outpatient clinic	Center for Epidemiologic Studies Depression Scale (CES-D) (score of 0–60, cut-off score for depression ≥ 16)	T1D T2D	36.24 (27.16)	2b/B
Alaqeel et al., [39], 2021, Saudi Arabia	Cross-sectional October 2020–April 2021	N = 148 Aged 8–16 years Female: 53.4% Duration of DM > 6 months	Outpatient clinic	Children’s Depression Inventory (CDI) (score of 0–54, ≥15: clinical depression symptoms)	T1D	Mild: 80% Moderate: 12.5% Severe: 7.5%	2b/B
Alkhormi et al., [50], 2022, Saudi Arabia	Cross-sectional August–February 2022	N = 375 Female: 51.7%	Diabetic center + primary healthcare centers	PHQ-9 (0–4: minimal, 5–9: mild, 10–14: moderate, 15–19: moderately severe, 20–27: severe depressive symptoms, cut-off score for depression >10)	T2D	Normal: 46% Moderate–severe: 54%	2b/B
Basit et al., [40], 2021, Pakistan	Cross-sectional August–September 2020	N = 380 Mean age: 51.93 (12.03) Female: 46.05%	Institute of diabetology and endocrinology	PHQ-9 (0–4: minimal, 5–9: mild, 10–14: moderate, 15–19: moderately severe, 20–27: severe depressive symptoms, cut-off score for depression >10)	T2D	None/minimal: 74.74% Mild: 22.63% Moderate: 2.63%	2b/B
Brown et al., [51], 2021, UK	Cross-sectional May–July 2020	N = 420 Mean age: 51.6 (9.9) Female: 87.8%	Online survey	PHQ-9 (0–4: minimal, 5–9: mild, 10–14: moderate, 15–19: moderately severe, 20–27: severe depressive symptoms, cut-off score for depression >10)	Obesity	13.4 (7) Minimal: 15.2% Mild: 20.5% Moderate: 27.6% Moderately severe: 22.4% Severe: 14.3%	2b/B
Celik et al., [52], 2021, Turkey	Cross-sectional	N = 142 Mean age: 53.7 Female: 45%	Outpatient clinic	HADS (range of 0–21, score of ≤ 7: normal, 8–10: mild, 11–14: moderate, ≥15: severe depression, cut-off score for depression ≥11)	Hypertension	6.35 (2.58) Moderate–severe: 18.3%	2b/B
Chao et al., [30], 2021, US	Longitudinal July–December 2020	N = 2829 Mean age: 75.6 (6) Female: 63.2%	Health center	PHQ-8 (0–4 minimal, 5–9 mild, 10–14 moderate, 15–19 moderately severe, 20–27 severe depressive symptoms, cut-off score for depression >10)	T2D	3.5 (4.0) None/minimal: 69.6% Mild: 21.9% Moderate: 6% Moderate–severe/severe: 2.5%	2b/B
Choudhary et al., [42], 2022, US	Longitudinal 2019–2020	N = 5732 Mean age: 13.8 (3.6) Female: 47.2%	Pediatric unit	PHQ-9 (0–4 minimal, 5–9 mild, 10–14 moderate, 15–19 moderately severe, 20–27 severe depressive symptoms, cut-off score for depression >10)	T1D	No difference between the 2019 and 2020 groups in PHQ-9 scores	2b/B
Cusinato et al., [45], 2021, Italy	Longitudinal March–April 2020	N = 117 Mean age: 15.9 (2.3) Female: 44% Mean duration of DM: 7.9 (4.6) years	Pediatric diabetes unit	Test of Depression and Anxiety Scale (TAD) (score of ≥115: clinical depressive symptoms)	T1D	16%	2b/B
D’Addario et al., [53], 2021, Italy	Longitudinal May–August 2020	N = 105 Mean age: 69.6 (5.8) Female: 39.4% Hypertension diagnosis >10 years: 76%	Telephone survey	HADS (range of 0–21, score of ≤ 7: normal, 8–10: mild, 11–14: moderate, ≥15: severe depression, cut-off score for depression ≥11)	Hypertension	3.1 (3.4)	2b/B
Deger et al., [54], 2021, Turkey	Longitudinal June–August 2020	N = 368 Age: 18–55 years Female: 78.8%	Outpatient clinic	PHQ-9 (0–4: minimal, 5–9: mild, 10–14: moderate, 15–19: moderately severe, 20–27: severe depressive symptoms, cut-off score for depression >10)	Obesity	Male: 15.26 (7.28) Female: 16.10 (7.44) Moderate–severe/severe: 60.1%	2b/B
Distaso et al., [55] 2022, UK	Cross-sectional January–March 2021	N = 369 Mean age: 50.5 (16) years Female: 52.9%	Diabetes clinics	PHQ-9 (0–4: minimal, 5–9: mild, 10–14: moderate, 15–19: moderately severe, 20–27: severe depressive symptoms, cut-off score for depression >10)	T1D T2D	7.28 (2–10) Moderate/moderate–severe/severe: 27.6%	2b/B
Durukan et al., [56], 2022, Turkey	Cross-sectional June–September 2020	N = 40 Mean age: 51.1 (6.9) Female: 22%	Outpatient clinic	HADS (range of 0–21, score of ≤ 7: normal, 8–10: mild, 11–14: moderate, ≥15: severe depression, cut-off score for depression ≥11)	Hypertension	6.5 (4.0) Mild: 22% Moderate: 12.5% Severe: 5%	2b/B
Kim & Kim, [46], 2022, Korea	Cross-sectional August–October 2020	N = 26695 Age > 19 years Female: 50.8% Mean duration of DM: > 5 years 57.5%	Nationwide health survey	PHQ-9 (0–4: minimal, 5–9: mild, 10–14: moderate, 15–19: moderately severe, 20–27: severe depressive symptoms, cut-off score for depression >10)	DM	Minimal/mild: 95.8% Moderate/moderately severe/severe: 4.2%	2b/B
Kim et al., [33], 2022, US	Cross-sectional June–December 2020	N = 84 Mean age: 68.46 (5.41) Female: 54.76% Mean duration of DM: 13.89 (7.53) years	Online survey	PROMIS—Depression (55: mild, 60: moderate, 65: moderately severe, 70: severe depression)	T2D	48.92 (8.10)	2b/B
Magliah et al., [47], 2021, Saudi Arabia	Cross-sectional June 2020	N = 65 Mean age: 30 (7.88) Female: 70.8% Mean duration of DM: 17.67 (6.89) years	Web survey	PHQ-9 (0–4: minimal, 5–9: mild, 10–14: moderate, 15–19: moderately severe, 20–27: severe depressive symptoms, cut-off score for depression >10)	T1D	None/minimal: 47.7% Mild: 29.2% Moderate: 15.4% Moderately severe: 6.2% Severe: 1.5%	2b/B
Moradian et al., [48], 2021, Germany	Cross-sectional April–June 2020	N = 253 Age ≥ 18 years Female: 74.3%	Online survey	PHQ-2 (ranges of 0–6, cut-off score for depression ≥3)	T1D T2D	Major depression symptoms Before COVID-19 outbreak: 11.9% After COVID-19 outbreak: 21.3%	2b/B
Musche et al., [29], 2021, Germany	Cross-sectional April–June 2020	N = 253 * Age > 18 years Female: 74.3%	Online survey	PHQ-2 (ranges of 0–6, cut-off score for depression ≥3)	T1D T2D	1.50 (1.75) Major depression symptoms T1D: 19.5% T2D: 25.7%	2b/B
Myers et al., [32], 2021, US	Longitudinal May–June 2020	N = 401 Mean age: 51.46 Female: 70.57% Mean duration of DM: 18.3 (9.9) years	Online survey	PHQ-8 (0–4: minimal, 5–9: mild, 10–14: moderate, 15–19: moderately severe, 20–27: severe depressive symptoms, cut-off score for depression >10)	T1D T2D	*T1D*7.99 (5.20) None/minima:l 21.98% Mild: 45.05% Moderate: 20.88% Moderately severe: 12.09% *T2D* 7.72 (5.92) None/minimal: 31.80% Mild: 32.16% Moderate: 19.08% Moderately severe: 16.96%	2b/B
Piskorz et al., [57], 2021, Latin American Countries	Cross-sectional June–July 2020	DM *n* = 899 Hypertension *n* = 3071 Dyslipidemia *n*= 1555 Age > 18 years	Online survey	DSM-5	DM Hypertension Dyslipidemia	DM 40% Hypertension: 37.64% Dyslipidemia: 38.52%	2b/B
Rechenberg & Koerner, [37], 2022, US	Cross-sectional	N = 146 Female: 42.2% Age: 13–17 years	Online survey	PHQ-2 (ranges of 0–6, cut-off score for depression ≥3)	T1D	2.75 (1.75)	2b/B
Sacre et al., [28], 2021, Australia	Longitudinal April 2020	N = 450 Mean age: 66 (9) Female: 31% Mean duration of DM: 12 years	Phone or online survey	PHQ-8 (0–4: minimal, 5–9: mild, 10–14: moderate, 15–19: moderately severe, 20–27: severe depressive symptoms, cut-off score for depression >10)	T2D	Pre-COVID-19 = 2.7 (2.4–3.0) Moderate/moderately severe/severe: 5.3% COVID-19 = 2.7 (2.4–3.0) Moderate/moderately severe/severe: 5.6%	2b/B
Sayed et al. [44], 2022, Egypt	Cross-sectional September 2020–June 2021	N = 403 Mean age: 46 (11.5) years Female: 59.1%	Primary care	PHQ-9 (0–4: minimal, 5–9: mild, 10–14: moderate, 15–19: moderately severe, 20–27: severe depressive symptoms, cut-off score for depression >10)	T2D	Moderate/moderate–severe/severe: 9.2%	2b/B
Shin et al. [58], 2021, Korea	Cross-sectional April–July 2020	N = 233 Mean age: 73.8 (5.7) Female: 59.3% Mean duration of DM: 17.7 (8.8) years	Outpatient clinic	PHQ-9 (0–4: minimal, 5–9: mild, 10–14: moderate, 15–19: moderately severe, 20–27: severe depressive symptoms, cut-off score for depression >10)	T2D	3.1 (3.6) Minimal: 73.7% Mild: 22% Moderate: 2.1% Moderate–severe: 2.1% Severe: 0%	2b/B
Silveira et al., [43], 2021, Brazil	Cross-sectional May–July 2020	N = 477 Mean age: 30.52 (9.22) Female: 83% Mean duration of DM: 15.29 (9.79) years	Web-based survey	PHQ-8 (0–4: minimal, 5–9: mild, 10–14: moderate, 15–19: moderately severe, 20–27: severe depressive symptoms, cut-off score for depression >10)	T1D	None/mild: 42.9% Moderate/moderate–severe/severe: 57.1%	2b/B
Sisman et al., [27], 2021, Turkey	Cross-sectional	N = 304 Mean age: 42.1 (15.5) Female: 56% Mean duration of DM: 10.3 (8.5) years	Web-based survey	HADS (score of ≤ 7: normal, 8–10: mild, 11–14: moderate, ≥15: severe depression, cut-off score for depression ≥11)	T1D T2D	6.2 (4) Mild/moderate/severe: 33.9%	2b/B
Souza et al., [41], 2021, Brazil	Cross-sectional April–May 2020	N = 162 Mean age: 42.5 (15.4) Female: 69.8%	Online survey	DASS-21 (score of 0–9: normal, 10–13: mild, 14–20: moderate, 21–27: severe, >27: extremely severe depression)	DM	Normal: 54.9% Mild: 12.3% Moderate: 12.3% Severe: 6.2% Extreme: 14.2%	2b/B
Tasnim et al., [31], 2021, Bangladesh	Cross-sectional November 2020–January 2021	DM *n* = 436 Hypertension *n* = 366 Obesity *n* = 193 Mean age: 42.29 (15.86) Female: 49.9%	Online survey	PHQ-9 (0–4: minimal, 5–9: mild, 10–14: moderate, 15–19: moderately severe, 20–27: severe depressive symptoms, cut-off score for depression >10)	DM Hypertension Obesity	DM 7.49 (6.53) Hypertension: 8.99 (6.76) Obesity: 9.62 (7.06)	2b/B
Wańkowicz et al., [36], 2021, Poland	Cross-sectional May 2020	N = 879 Age > 18 years	Inpatient units and outpatient clinics	PHQ-9 (0–4: minimal, 5–9: mild, 10–14: moderate, 15–19: moderately severe, 20–27: severe depressive symptoms, cut-off score for depression >10)	Hypertension DM Dyslipidemia	Hypertension: 11.51 (5.45) DM: 11.80 (4.61) Dyslipidemia: 12.08 (5.73)	2b/B
Yazıcı et al., [59], 2022, Turkey	Longitudinal March 2020	N = 422 Mean age: 45 (12.7) Female: 84%	Online survey	PHQ-9 (0–4: minimal, 5–9: mild, 10–14: moderate, 15–19: moderately severe, 20–27: severe depressive symptoms, cut-off score for depression >10)	Hypertension	7.7 (5.9) Moderate/severe: 28.4%	2b/B

* Same sample as Moradian et al. 2021. DASS-21 = Stress, Anxiety and Depression Scale; DM = diabetes mellitus; DSM-5 = *Diagnosis Manual of Mental Disorders* fifth edition; EL = evidence level; HADS = Hospital Anxiety and Depression Scale; IG = intervention group; IQR = interquartile range; PHQ-2= Patient Health Questionnaire—2; PHQ-8 = 8-item Patient Health Questionnaire; PHQ-9 = 9-item Patient Health Questionnaire; PROMIS = Patient-Reported Outcomes Measurement Information System; RG = recommendation grade; T1D = type 1 diabetes; T2D = type 2 diabetes; SD = standard deviation.

### 3.3. Levels of Depression and Related Factors in Patients with other Chronic Pathologies

In patients with obesity, levels of depression ranged from mild [31] to moderate [51] to moderate–severe [54]. An association has been found between depression and a higher frequency of unhealthy diets (OR = 1.06, 95% CI = 1.02–1.11, *p* = 0.003) and between depression and a lower quality of life (OR = 0.97, 95% CI = 0.95–0.99, *p* = 0.010) [51]. Moreover, depression was inversely associated with physical activity (OR = 1.05, 95% CI = 1.01–1.10, *p* = 0.019) [51] (Table 2).

Among patients with hypertension, the reported levels of depression ranged from minimal [52,53,56] to mild [31,59] to moderate [36]. Depression was also related to increased blood pressure (*p* < 0.001) [52] and psychological distress [53] (Table 2).

Among the few studies to have considered the relationship between depression and dyslipidemia, a moderate association was reported [36] (Table 2).

**Table 2:** Main predictors of depressive symptoms and their correlations with chronic diabetes.

Author (Year)	Predictors and Correlates of Depressive Symptoms among DM Patients
Abdelghani et al. (2021) [34]	Poor physical component summary (OR = 0.88, 95% CI = 0.78–0.99, *p* = 0.045) Poor mental component summary (OR = 0.84, 95% CI = 0.74–0.96, *p* = 0.009)
Abdoli et al. (2021) [38]	Female gender (OR = 1.83, 95% CI = 1.26–2.66, *p* = 0.0008) Younger (OR = 1.02, 95% CI = 1.01–1.04, *p* < 0.0001) Single (OR = 1.47, 95% CI = 1.09–1.97, *p* = 0.014) Education level (OR = 2.74, 95% CI = 1.59–4.73, *p* = 0.0032) Higher HbA1c (OR = 1.26, 95% CI = 1.12–1.43, *p* < 0.000) Lower daily time-in-range blood glucose (OR = 1.01, 95% CI = 1– 1.02, *p* = 0.0002) Difficulties accessing healthy food (OR = 1.39, 95% CI = 1.05–1.85, *p* = 0.019) Changes in diabetes self-care behaviors (OR = 1.70, 95% CI = 1.27–2.27, *p* < 0.0001) Fear of approaching diabetes facilities (OR = 1.33, 95% CI = 1.05–1.77, *p* = 0.0458)
Alaqeel et al. (2021) [39]	Female sex (aOR = 4.55, 95% CI = 1.80–11.48, *p* = 0.001) Uncontrolled HbA1c level (aOR = 7.12, 95% CI = 1.93–26.32, *p* = 0.003) Longer diabetes duration (DM duration of ≥ 5 years aOR = 4.82, 95% CI = 1.07–21.65, *p* = 0.040)
Ajele et al. (2022) [35]	Direct relationship between depression and psychological well-being (β = 36, *p* < 0.05) Negative relationship between depression and diabetes distress (β = −0.47, *p* < 0.05)
Basit et al. (2021) [40]	Fear of COVID-19 (OR = 4.68, 95% CI = 0.96–22.68, *p* = 0.05)
Chao et al. (2021) [30]	Female sex (aOR = 1.4, 95% CI = 1.1–1.7) Obesity (aOR = 1.3, 95% CI = 1.0–1.5)
Choudhary et al. (2022) [42]	Female (*p* < 0.0001)
Cusinato et al. (2021) [45]	Lower time in glucose range (*p* = 0.012)
Kim & Kim (2022) [46]	Decreased physical activity (aOR = 1.34, 95% CI = 1.15–1.55) Decreased sleep time (aOR = 1.87, 95% CI = 1.56–2.24) Increased junk food or carbonated beverages consumption (aOR = 1.48, 95% CI = 1.11–1.99) Increase in frequency of food delivery consumption (aOR = 1.54, 95% CI = 1.15–2.08) Increased alcohol consumption (aOR = 2.46, 95% CI = 1.62–3.71) Increase in cigarette consumption (aOR = 1.92, 95% CI = 1.27–2.90)
Kim et al. (2022) [33]	Worries associated with COVID-19 and depression score (r = 0.46, *p* = 0.000)
Moradian et al. (2021) [48]	COVID-19-related fear (*p* < 0.001)
Musche et al. (2021) [29]	COVID-19-related fear (*p* = 0.006)
Myers et al. (2021) [32]	Female (*p* < 0.001) Youngest age group 18–34 years (*p* < 0.001)
Rechenberg & Koerner, (2022) [37]	Poorer general treatment-related quality of life (*p* < 0.001)
Sayed et al. (2022) [44]	Higher HbA1c level (*p* < 0.05)
Silveira et al. (2021) [43]	Difficulty accessing diabetes supplies (*p* < 0.05) Higher HbA1c level (*p* < 0.05)
Sisman et al. (2021) [27]	Individuals with T2D (*p* = 0.03)
Souza et al. (2021) [41]	Female sex (OR = 2.5, 95% CI = 1.33–4.72, *p* = 0.004) Single (OR = 4.1, 95% CI = 2.34–7.13, *p* < 0.001) No religion (OR = 2.2, 95% CI = 1.34–3.54, *p* = 0.002) History of anxiety and/or depression (OR = 2.6, 95% CI = 1.64–4.14, *p* < 0.001) Reduced monthly income during the pandemic period (OR = 1.9, 95% CI = 1.18–3.11, *p* = 0.008) Reduced work or remote study (OR = 1.9, 95% CI = 1.18–3.11, *p* = 0.008)
Wańkowicz et al. (2021) [36]	Female sex (*p* = 0.013)

AOR = adjusted OR, DM = diabetes mellitus, T2D = type 2 diabetes.

### 3.4. Meta-Analysis

**Figure 2:**
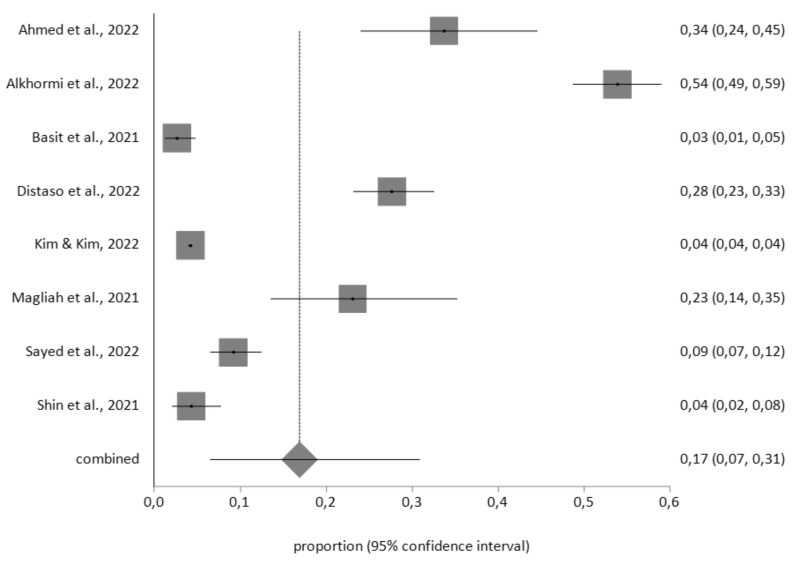
Prevalence of depression in diabetic patients determined using the Patient Health Questionnaire—9 items (PHQ-9).

**Figure 3:**
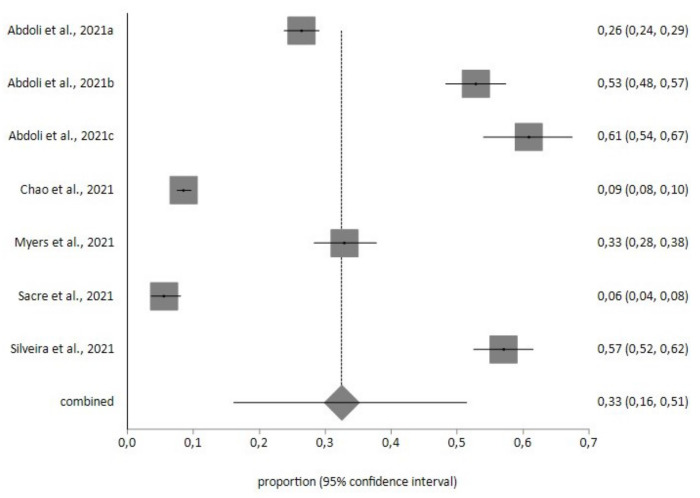
Prevalence of depression in diabetic patients determined using the Patient Health Questionnaire—8 items (PHQ-8).

Four meta-analyses (three random-effects and one fixed-effect) were performed, with a total study population of 34554 patients with diabetes, 788 with obesity and 182 with hypertension. In the diabetic patients, the prevalence of moderate, moderately severe and severe levels of depression (cutoff score >10), according to the PHQ-9 instrument (*n* = 28609), was 17% (95% CI = 7–31), with a high level of heterogeneity (I^2^ = 98%), and Egger’s test: *p* = 0.593 (see Figure 2). For the PHQ-8 instrument, with a total sample of *n* = 5945, the prevalence of depression was 33% (95% CI = 16–51) with high heterogeneity (I^2^ = 99.5%), and Egger’s test: *p* = 0.532 (see Figure 3).

**Figure 4:**
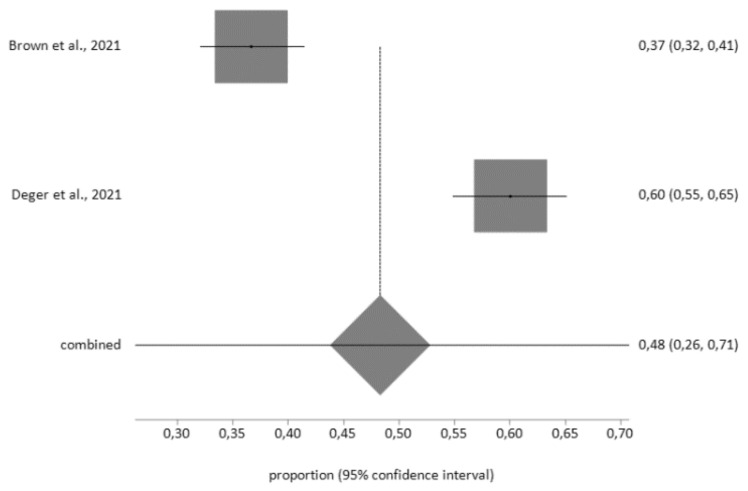
Prevalence of depression in obesity patients determined using the Patient Health Questionnaire—9 items (PHQ-9).

In patients with obesity, the prevalence of depression, according to the PHQ-9 questionnaire, was 48% (95% CI = 26–71) for moderately severe and severe levels (score >15 points), with high heterogeneity (I^2^ = 97.7 %), and Egger’s test: *p* = 0.132 (see Figure 4).

**Figure 5:**
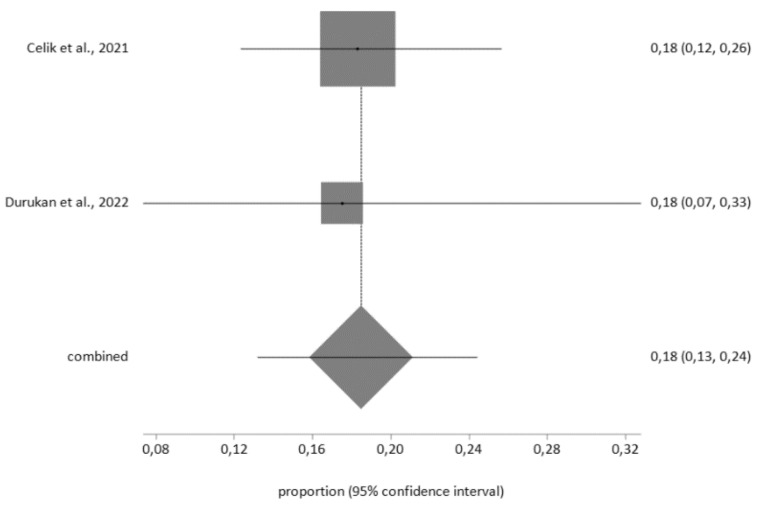
Prevalence of depression in hypertensive patients using the Hospital Anxiety and Depression Scale (HADS).

For hypertensive patients, the prevalence of depression measured using the HADS questionnaire was 18% (95% CI = 13–24) for moderate, moderately severe and severe levels of depression (cutoff score >10) with low heterogeneity (I^2^ = 0%), and Egger’s test: *p* = 0.213 (see Figure 5).

For all four meta-analyses, Egger’s test revealed no publication bias. The sensitivity analysis showed every meta-analysis to be satisfactory in this respect.

## 4. Discussion

In the studies considered, the prevalence of depression among diabetic patients ranged from 17% (PHQ-9) to 33% (PHQ-8). Although the sensitivity and specificity for both questionnaires in detecting major depression are similar [60], some studies propose PHQ-8 for use in the medical population due to the controversial “suicide item”(item-9) on PHQ-9 [61,62]; this fact could explain the difference in prevalence. For patients with obesity, depression was 48%, and it was 18% for hypertensive patients. The differences in prevalence among chronic patients can be attributed to the differences in personality factors, symptom burden, adherence to self-care regimens, and lifestyle, culture or healthcare systems [63].

Additionally, the number of comorbid conditions is significantly associated with major depression. Some authors showed that depression prevalence ranged from 6.7% in those with diabetes alone to 17% in those with diabetes and three or more additional coexisting chronic conditions (e.g., hypertension or obesity) [64]. Another study among adults with T2D and hypertension and obesity indicated a prevalence greater than 18% for severe depression among T2D respondents with comorbidities [65]. However, not all the studies included in our review indicated the type of concomitant chronic disease or the percentage of subjects with those comorbidities, so we have not been able to establish a relationship between the prevalence of depression related to each comorbidity.

Prior to the pandemic, the prevalence of depression in patients with type 2 diabetes ranged from 10.6% to 17% [66,67]. In children and patients with type 1 diabetes, it was 30% [68], 23% in obese patients [69] and 14.5% in hypertensive patients [70]. These data provide evidence that the prevalence of depressive symptoms increased during the pandemic [71]. Although some studies of the general population [8,72,73] have reported a prevalence of depression of up to 20.3% at the start of the COVID-19 pandemic [74], others have recorded even higher values compared to pre-pandemic rates (27.8% vs. 8.5%) [8,75].

Chronic disease increases the risk of the patient suffering an emotional and/or behavioral disorder, especially in times of social crisis such as that experienced with the COVID-19 pandemic [76]. This relationship has been demonstrated, with different levels of intensity and frequency, for the signs and symptoms of depression [77]. Indeed, depression may even be a risk factor for suicide in chronically ill patients [78].

With respect to related factors, studies of diabetic patients have highlighted the following as significant predictors of depression: being single, having a low income, having a relatively low level of education and being a smoker. These relationships have been observed in studies performed both before and after the pandemic [8,66,79]. Another factor associated with depression is that of gender. Thus, previous studies have reported higher rates of depressive symptoms in women than men [66,71,74,80,81,82]. Among patients with type 1 diabetes, depressive symptoms are present in 17.5% of women but in only 8.6% of men. For those with type 2 diabetes, the corresponding figures are 28.9% and 19.8% [83].

Clinical parameters related to higher levels of depression in diabetic patients include elevated HbA1c, lower daily time-in-range blood glucose, long-course diabetes and sleep disorders [66,83,84,85]. On the other hand, previous studies conducted before the pandemic found no relationship between HbA1c and levels of depression [67].

Our study did not reveal a clear relationship between age and depression. Thus, some studies have reported worse mental health among young people [74,81,86,87], while others have observed higher rates of depression in older age groups [83,88]. The existence of obesity as a predisposing factor is also inconsistent, although some authors associate a worse mental state and greater stress with unhealthy patterns of behavior [66].

Previous levels of anxiety and depression are predictors of depression for patients with diabetes, and some authors have also included loneliness as a predisposing factor for its development [89,90,91,92,93].

One of the main significant predictors of depression is the fear of contagion with COVID-19. Thus, during the pandemic, many chronic patients did not attend hospital for attention [3] and only half were willing to do so even in the event of an emergency [94].

Another factor related to the greater vulnerability to depression of chronic patients is their lack of adherence to disease management. Up to a third of these patients failed to adhere completely to their pharmacological treatment program [2], and many increased their consumption of sugary foods and snacks and were physically inactive, especially during the lockdown period [95]. These circumstances provoked worsening of the general state of health in 52% of adults and 38% of children with chronic disease [96,97].

Feelings of social disconnection and of a lack of communication with health professionals may have hindered access to primary care, which, in turn, produced discontinuity of care [98]. According to other studies, up to 59% of patients wished they had received additional information about the risks associated with their medical condition during the pandemic [2]. Patients’ fear of re-entering society after pandemic-induced isolation has also been related to psychological problems and difficulties such as hallucinations, which are often underestimated or not recognized [8].

Few studies have analyzed rates of depression and related factors in order to identify and distinguish the mental health consequences of exposure to the COVID-19 pandemic. In consequence, much remains to be known about mental health care for the general population and for chronic patients in particular. Furthermore, the management of chronic diseases in the midst of the COVID-19 pandemic has been especially challenging, raising a multitude of questions about factors such as susceptibility to infection, the incidence of complications and problems related to the continuation of maintenance treatment. In consequence, it is essential to further analyze the existing levels of depression and related risk factors in order to enhance the psychological state of patients with chronic diseases.

### 4.1. Limitations

This study presents various limitations that must be acknowledged. First, most of the studies included were cross-sectional, which limits their ability to infer causal relationships. Second, the study data were collected over different periods, which may have distorted the values obtained for depression among the population (for example, the successive waves of the pandemic would have led to varying effects over time). In consequence, there is inevitable heterogeneity in the results presented. Third, methodological differences in the sampling and recruitment strategies employed to collect data (for example, the use of online survey vs. face-to-face surveys) may have introduced bias into the findings reported. Fourth, although the prevalence of depression was lower in patients with hypertension, our search included only a limited number of chronic conditions, so these results should be interpreted with caution. Finally, although all the studies included in our review used a validated measurement tool as a good screening instrument for depression [60], some of them, such as the HADS, do not include all the items of the diagnostic criteria for depression as described in the DSM-IV or ICD-10; these include necessary additional questions about appetite, sleep or self-harm/suicidal thoughts [99]. This may lead to failure to identify a sufficiently accurate threshold score for classifying patients with depression.

To overcome these limitations, future research in this area should take the form of large-scale longitudinal studies, including analyses of a larger number of chronic diseases.

### 4.2. Implications for Practice and Research

The COVID-19 pandemic has severely aggravated levels of depression among patients with chronic disease, which highlights the need for early psychological intervention to address their mental health needs, since the numbers affected will continue to rise [100]. In addition, further research is needed to consider the risk factors associated with individual diseases, and to continue analyzing possible mental health disorders in these patients, which may differ in type and prevalence from those existing prior to the pandemic.

Self-care and adherence to treatment are essential for patients with chronic disease; therefore, access to primary care should be prioritized and facilitated, for example, through the development of new channels for this purpose [91]. In this respect, telemedicine has proven to be beneficial in ensuring continuity of care and disease monitoring. This and other types of intervention could be useful if incorporated into mental health attention and routine care for chronic patients [94,101]. Another valuable area for future interventions would be to encourage patients and healthcare professionals to become more proactive in digitization, thus enhancing health services and personalizing medical care [102,103].

## 5. Conclusions

The COVID-19 pandemic has had a severe impact on many areas of public health. One such impact has been on patients with chronic illnesses, many of whom have suffered increased levels of depression. However, with the use of validated depression tools based on statistical criteria, the broad clinical variability could make it difficult to diagnose depression levels; therefore, the levels of depression found must be interpreted with caution.

Far-reaching events such as the pandemic have major repercussions on mental health, and specific healthcare policies are needed to assure the physical and mental health of chronic patients. Interventions to reduce the potential psychological harm caused by the COVID-19 pandemic are especially needed.

## Data Availability

Not applicable.

## Supplementary Materials

The following supporting information can be downloaded at: https://www.mdpi.com/article/10.3390/diagnostics12123094/s1, Supplementary Material S1: PRISMA 2020 checklist.
